# *EGFR* mutation status in tumour-derived DNA from pleural effusion fluid is a practical basis for predicting the response to gefitinib

**DOI:** 10.1038/sj.bjc.6603428

**Published:** 2006-10-24

**Authors:** H Kimura, Y Fujiwara, T Sone, H Kunitoh, T Tamura, K Kasahara, K Nishio

**Affiliations:** 1 Shien-Lab, National Cancer Center Hospital, Tsukiji 5-1, Chuo-ku, Tokyo, Japan; 2 Respiratory Medicine, Kanazawa University Hospital, Takara-machi13-1, Kanazawa, Ishikawa, Japan; 3 Medical Oncology, National Cancer Center Hospital, Tsukiji 5-1, Chuo-ku, Tokyo, Japan; 4 Pharmacology Division, National Cancer Center Research Institute, Tsukiji 5-1-1, Chuo-ku, Tokyo, Japan; 5 Center for Medical Genomics, National Cancer Center Research Institute, Tsukiji 5-1-1, Chuo-ku, Tokyo, Japan

**Keywords:** pleural effusion, EGFR, mutation, gefitinib

## Abstract

*Epidermal growth factor receptor (EGFR)* mutations are strong determinants of tumour response to EGFR tyrosine kinase inhibitors in non-small-cell lung cancer (NSCLC). Pleural effusion is a common complication of lung cancer. In this study, we assessed the feasibility of detection of *EGFR* mutations in samples of pleural effusion fluid. We obtained 43 samples, which was the cell-free supernatant of pleural fluid, from Japanese NSCLC patients, and examined them for *EGFR* mutations. The *epidermal growth factor receptor* mutation status was determined by a direct sequencing method (exons 18–21 in *EGFR*). *EGFR* mutations were detected in 11 cases (E746_A750del in seven cases, E746_T751del insA in one case, L747_T751del in one case, and L858R in two cases). The *EGFR* mutations were observed more frequently in women and non-smokers. A comparison between the *EGFR* mutant status and the response to gefitinib in the 27 patients who received gefitinib revealed that all seven patients with partial response and one of the seven patients with stable disease had an *EGFR* mutation. No *EGFR* mutations were detected in the patients with progressive disease. The results suggest that DNA in pleural effusion fluid can be used to detect *EGFR* mutations and that the *EGFR* mutation status may be useful as a predictor of the response to gefitinib.

Lung cancer is a major cause of cancer-related mortality worldwide and is expected to remain a major health problem for the foreseeable future (Parkin *et al*, 2005). Most patients have advanced disease at the time of diagnosis. The initial therapy for advanced non-small-cell lung cancer (NSCLC) is systemic chemotherapy with a two-drug combination regimen, which often includes a platinum agent, but the median survival of patients treated with such regimens has ranged from only 8 to 10 months. Little improvement in the efficacy of chemotherapy has been made in the last 20 years (Breathnach *et al*, 2001; Kelly *et al*, 2001; Schiller *et al*, 2002).

Targeting epidermal growth factor receptor (EGFR) is an appealing strategy for the treatment of NSCLC, because EGFR has been found to be expressed, sometimes strongly, in NSCLC (Franklin *et al*, 2002). Gefitinib (‘Iressa’, AstraZeneca) is a small molecule and selective EGFR tyrosine kinase inhibitor that has shown antitumour activity in NSCLC patients as a single agent in phase II trials (Fukuoka *et al*, 2003). Adding gefitinib to chemotherapy in phase III studies of patients with untreated advanced NSCLC did not significantly improve the outcome over chemotherapy alone (Giaccone *et al*, 2004; Herbst *et al*, 2004), and a possible explanation for the failure to observe any added benefit in these trials is that the patients had not been screened or selected for their ability to derive any clinical benefit from an EGFR inhibitor.

An association between mutations in sites of *EGFR* tyrosine kinase in NSCLC and hyper-responsiveness to gefitinib has recently been reported (Lynch *et al*, 2004; Paez *et al*, 2004). The mutations consisted of small in-frame deletions or substitutions clustered around the ATP-binding site in exons 18, 19, and 21 of *EGFR*, and the mutations increased the affinity of the enzyme for ATP and gefitinib (Lynch *et al*, 2004). Some investigators subsequently found that *EGFR* mutations are one of the strong determinants of tumour response to EGFR tyrosine kinase inhibitors (Pao *et al*, 2004; Han *et al*, 2005). The investigators used surgical tissue to detect the *EGFR* mutations in their studies, but most patients who require gefitinib therapy are diagnosed at an advanced stage of the disease and are inoperable. As it is often difficult to obtain a sufficient tumour sample from patients with inoperable NSCLC to detect *EGFR* mutations by direct sequencing, a method of detecting *EGFR* mutations in other specimens needed to be established.

Malignant pleural effusion is a common complication of lung cancer. It is present in approximately 15% of patients at the time of diagnosis (Pass *et al*, 2005) and in 10–50% of patients during the course of the disease (Fenton and David Richardson, 1995). In about half of NSCLC patients with a pleural effusion, the effusion fluid is cytologically positive at the first time examined, and ultimately most effusions are determined to be malignant. As pleural effusion fluid sampling is usually easy, non-invasive, and repeatable, we hypothesised that tumour-derived DNA in the pleural effusion fluid of NSCLC patients would be a source of useful information on the status of the *EGFR* gene and could allow prediction of the response to gefitinib. Some investigators have reported that pleural effusion fluid is a useful clinical specimen for searching for point mutations in oncogenes, such as *K-ras*, *rho A*, *p53*, and *FHIT* (Nakamoto *et al*, 2001; Lee *et al*, 2004). As the two trials were small, the results regarding the sensitivity and specificity of detection of the mutations in pleural effusion as a diagnostic method were unclear. Detection of *EGFR* mutations in pleural effusion fluid has been described in one case report, and the patient responded to gefitinib (Huang *et al*, 2005). The results in that patient encouraged us to hypothesise that the *EGFR* mutation status determined in pleural effusion fluid is useful for predicting the responsiveness to EGFR tyrosine kinase inhibitors.

In the present study, we attempted to detect *EGFR* mutations in pleural effusion fluid and to clarify the usefulness of their detection as a predictor of the response to gefitinib.

## PATIENTS AND METHODS

### Patients

The subjects were NSCLC patients who had a pleural effusion at the time of diagnosis. The diagnosis of NSCLC was based on the histological or cytological findings, and the histological type was determined according to the WHO criteria (Travis *et al*, 1999). Patients' records consisted of age, gender, smoking habit, histological type, and treatment. Smoking status was collected from the patients' records. Patients were divided into three groups according to their smoking status: never smokers (<100 cigarettes/lifetime), former smokers (⩾100 cigarettes/lifetime, no smoking at present), and current smokers (⩾100 cigarettes/lifetime). The response of the patients treated with gefitinib was evaluated every 4 or 8 weeks in accordance with the ‘Response Evaluation Criteria in Solid Tumours (RECIST)’ guidelines. (Therasse *et al*, 2000). Partial response (PR) and stable disease (SD) were confirmed by a sustained 4-week follow-up. This study was approved by the Institutional Review Board of the National Cancer Center Hospital and of Kanazawa University Hospital, and written informed consent was obtained from all participants. No research results were entered into the patient's records or released to the patient or the patient's physician.

### Collection of pleural effusion fluid and DNA purification

The pleural effusion fluid was collected into heparinised tubes between 29 March 2005 and 30 January 2006. No particular collection method was used. A 2-ml sample of the fluid was centrifuged at 250 *g* for 10 min at room temperature, and the supernatant was collected and stored at −80°C until DNA extraction. DNA was extracted from 1 ml of the supernatant with a Qiamp DNA Mini Kit (Qiagen, Hilden, Germany) according to the blood and body fluid spin protocol in the manufacturer's instructions, with the following protocol modifications. The same column was used repeatedly until the whole sample had been processed. The DNA obtained was eluted in 50 *μ*l of sterile bi-distilled buffer, and the extracted DNA was stored at −20°C until used. The amounts of DNA extracted were estimated with spectrophotometry.

### Polymerase chain reaction amplification and direct sequencing

Exons 18, 19, 20, and 21 of the *EGFR* gene were amplified by polymerase chain reaction (PCR). The primers were designed based on the report by Lynch *et al* (2004). Genomic PCR of 1 *μ*l of template DNA was performed in 25 *μ*l volumes containing 0.75 U of Ampli Taq Gold DNA polymerase (Perkin-Elmer, Roche Molecular Systems Inc., Branchburg, NJ, USA), 2.5 *μ*l of PCR buffer, 0.8 *μ*M dNTP, 0.5 *μ*M of each primer, and different concentrations of MgCl_2_, depending on the polymorphic marker. The first PCR analyses were performed in a volume of 25 *μ*l by 25 cycles consisting of a denaturation step at 94°C for 45 s, a primer annealing step at 58°C for 30 s, and an elongation step at 72°C for 30 s. The final step at 72°C was extended for 10 min. Nested PCR was performed with 20 cycles under the same conditions as the first PCR. Sequencing of each sample was performed in duplicate with an ABI prism 310 (Applied Biosystems, Foster City, CA, USA). PCR products were sequenced in both sense and antisense directions. *Epidermal growth factor receptor* mutations detected in the initial round of sequencing were confirmed by subsequent rounds of independent PCR and sequencing reactions. Only specimens in which a mutation was identified in both rounds were recorded as mutation-positive. The sequences were compared with the GenBank-archived human sequence for *EGFR* (accession number: AY588246). The nucleic acid and protein coordinates used to name the mutations are based on NM_005228.3 and NP_005219.2, respectively.

### Statistical analyses

This study was carried out as exploratory research for detecting *EGFR* mutations from pleural effusion fluid and clarifying the relationship between the mutation status and clinical manifestations. The number of enrolled patients was therefore not precalculated. Patient characteristics, including gender, tumour histology, and smoking habit were tabulated according to their mutation status. Fisher's exact test was used to test for associations between the presence of *EGFR* mutations and the patients' characteristics. The relationship between response to gefitinib and the mutation status was evaluated individually.

## RESULTS

### Patients and pleural effusion specimens

Forty-three patients were enrolled in this study (Table 1). Two hundred and sixty-two patients were seen with stage IIIB and IV at our institutions in the period of this study. Forty-three of the 262 patients were enrolled in this study. The enrolled patients were not all of the patients with pleural effusion because written informed consent was not obtained from any patients with pleural effusion. Their median age was 62 years (range, 39–82 years), and there were 21 females (53.8%) and 17 never smokers (43.6%). The histological and/or cytological diagnosis was adenocarcinoma in 39 patients, and squamous cell carcinoma and large cell carcinoma in one each, and unclassified NSCLC in two patients. Non-small-cell lung cancer cells in the pleural effusion samples of 40 of the patients were identified cytologically. There were no malignant cells in the pleural effusion fluid of the other three patients. We have no data of the proportion of malignant cells and normal cells. Twenty-seven patients were treated with gefitinib (250 mg day^−1^) and evaluated for a response. Eight of the 27 patients were treated with gefitinib as an initial treatment and the other 19 patients were treated with the agent as a second or third line. The others were treated with systematic chemotherapy, including a platinum agent. The results of the evaluation showed that seven of the 27 patients who received gefitinib therapy had a PR and seven had SD. The other 13 patients had progressive disease (PD). DNA was extracted from all 43 samples of pleural effusion fluid. Amounts of the DNA extracted were detectable from 27 samples at a concentration up to 144.0 ng ml^−1^. Amounts from 16 samples were under the detectable limit.

### Detection of *EGFR* mutations in pleural effusion fluid

**Figure 1:**
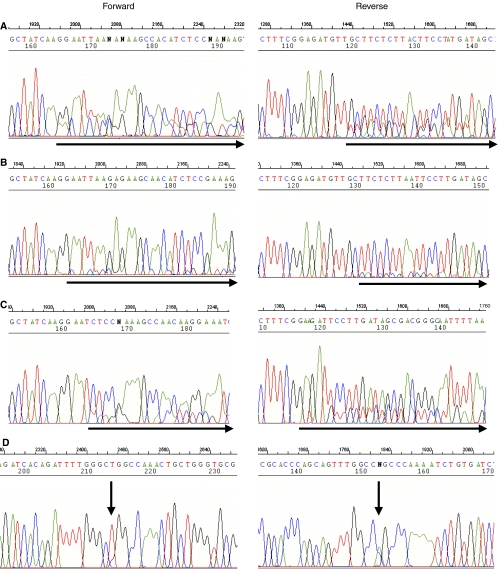
The wave figures of the nucleotide sequence of the *EGFR* gene with heterozygous mutations obtained by direct sequencing (see ‘Patients and Methods’) are shown. Horizontal arrows in both the sense and the antisense directions are shown to demonstrate the two breakpoints of the deletion. The patients in **A**, **B**, and **C** have inframe deletions in exon 19 (Figure **A**, E746_A750del; **B**, E746_T753del insA; **C**, L747_T751del; **D**, L858R). The double peaks (vertical arrows) represent the heterozygous missense mutations resulting in an amino acid substitution of L858R in exon 19 (Figure **D**).

Direct sequencing of PCR products in exons 18–21 of *EGFR* in the pleural effusion fluid of all patients allowed their mutation status to be determined. Heterozygous mutations were identified in 11 (25.6%) of the 43 patients (Table 1). Nine mutations were deletional mutations located in exon 19 (E746_A750del in seven, L746_T751del insA in one, L747_T751del in one), and two were substitution mutations located in exon 21 (L858R) (Table 2) (Figure 1). No mutations were detected in exon 18 or 20. The E746_A750 deletion and L858R substitution mutations were the most common (9 out of 11 mutations. 81.8%) and are well-known hotspot mutations described previously (Kosaka *et al*, 2004; Pao *et al*, 2004). No more than one mutation was identified per patient, and no EGFR mutations were detected in pleural effusion fluid that did not contain malignant cells.

### *Epidermal growth factor receptor* mutation status and patients' characteristics

*EGFR* mutations were detected more frequently in the samples from females (7 out of 21, 33.3% of females, 4 out of 18, 22.2% of males; *P*=0.310) and non-smokers (7 out of 17, 41.1% of non-smokers, 4 out of 22, 18.1% of current or former smokers; *P*=0.156), although the differences were not statistically significant (Table 3). Of the 11 mutations, 63.6% were in women and 63.6% were in non-smokers. All of the patients with mutations had adenocarcinoma. No *EGFR* mutations were found in any of the patients with squamous carcinoma or large cell carcinoma. A comparison between the *EGFR* mutant status and the response to gefitinib showed that all seven patients with a PR and one of the seven patients with SD had an *EGFR* mutation. No *EGFR* mutations were detected in any of the patients with PD (Table 4). We have no response data from the 16 patients who had never treated with gefitinib, and we have not evaluated the relationship between the response to chemotherapy and the EGFR mutation status in pleural effusion fluid.

## DISCUSSION

This is the first report of an analysis of the *EGFR* mutation status in DNA obtained from the pleural effusion fluid of a series of NSCLC patients and evaluation of the relationship between the mutation status and the clinical response to gefitinib. It is interesting that all patients who achieved a PR to gefitinib had the *EGFR* mutations. We hypothesised that the mutation status in DNA extracted from pleural effusion fluid would allow prediction of the clinical outcome of gefitinib therapy in NSCLC patients, and we therefore expected the pleural effusion fluid to be a practical source of DNA for detection of *EGFR* mutations. The sites of *EGFR* mutations found in this study are identical to these reported in previous studies (Kosaka *et al*, 2004; Pao *et al*, 2004). The main mutations found were in-frame deletions in exon 19 and the missense mutation L858R in exon 21. No patients had more than one mutation. It was possible to determine the mutation status of *EGFR* by using the DNA in only 1.0 ml of pleural effusion fluid, even though the concentration of the extracted DNA specimens was in most cases below the concentration detectable by spectrophotometry (data not shown). The results of the comparison between the mutation status and clinical manifestations in this study confirmed the finding in previous studies that *EGFR* mutations are frequently present in small sub-groups of NSCLC patients, such as females and never smokers, although the differences were not statistically significant. It is well known that *EGFR* mutations are frequently observed in adenocarcinomas. As 36 of the 39 patients (92.3%) enrolled in this study had adenocarcinoma, we could not evaluate differences in the frequency of the *EGFR* mutations according to the histological type. Pleural effusion occurs in lung carcinoma of all cell types, but appears to be most frequent in adenocarcinoma (Chernow and Shahn, 1997).

This study had several limitations. First, we could not compare the results of the *EGFR* mutation status in the pleural effusion fluid to the mutation status in tumour tissue. Forty of the 43 patients enrolled were cytologically diagnosed as having NSCLC from pleural effusion fluid specimens. As the DNA extracted from pleural effusion fluid consisted of DNA derived from both tumour cells and normal cells, the *EGFR* mutation status needs to be evaluated in a pair of DNA specimens from the tumour and pleural effusion fluid to confirm the usefulness of the mutation status determined from pleural effusion fluid. However, it is sometimes difficult to obtain tumour samples from patients with advanced NSCLC, and even more so from patients diagnosed as having NSCLC using methods other than the histological examination of tumour tissue, such as on the basis of pleural effusion or sputum cytology. Second, direct sequencing may be not able to provide satisfactory results for detection of *EGFR* mutations in mixed samples of mutated and wild DNA. Although direct sequencing has generally been used to detect *EGFR* mutations in previous studies, detection of a mutation by this method requires at least 30% of the mutated DNA in a sample (Bosari *et al*, 1995; Fan *et al*, 2001). Lung cancers are very heterogeneous, and as normal cells, such as inflammatory cells or mesothelial cells, are contained in the pleural effusion fluid of lung cancer patients, in addition to tumour cells, a small amount of mutated DNA in pleural effusion fluid can be missed by direct sequencing. Unfortunately, we have no data at the present time on whether EGFR mutations were detectable in pleural effusion samples with either a few malignant cells, a small proportion of malignant cells with normal mesothelial cells, or cytologically negative samples. To establish a method for the detection of EGFR mutations from pleural effusion fluid, the mutation detectable proportion of malignant cells to normal cells in pleural fluid should be elucidated. We are planning an additional study using cytological examination to clarify the mutation detectable proportion as a next step. When pleural fluid is used as the material for detection of *EGFR* mutations, a patient with an *EGFR* mutation may be diagnosed as having wild-type *EGFR* because of the two limitations described above. Although we expected a high frequency of detection of *EGFR* mutations in this study because of the high proportion of adenocarcinomas (92.3%), we detected *EGFR* mutations in only 28.2% of the patients enrolled, a lower frequency than in two previous reports on Japanese NSCLC patients (Takano *et al*, 2005; Asano *et al*, 2006). Patients with false-negative results, meaning that no *EGFR* mutations were detected in a patient with an *EGFR* mutation, were not excluded from this study. Some investigators have tried to improve the sensitivity of detection of *EGFR* mutations in samples containing a mixture of tumour and normal cells. Wookey *et al* (2005) reported findings that the ARMS method was superior to the direct sequencing method and WAVE method for detecting *EGFR* mutations. Other groups have reported that LightCycler PCR assay (Sasaki *et al*, 2005), SSCP assay (Marchetti *et al*, 2005), and enriched PCR assay (Asano *et al*) are more sensitive than direct sequencing and are more rapid. A standardised method of detecting *EGFR* mutations needs to be established as soon as possible.

The final limitation in the present study is that it remains unclear whether there is any survival benefits associated with gefitinib therapy in those patients enrolled with *EGFR* mutations. The relationship between the *EGFR* mutation status determined in pleural effusion fluid and the gefitinib response in a portion of the patients enrolled supports the pleural effusion fluid *EGFR* mutation status as useful for predicting the response to gefitinib. The relationship between the *EGFR* mutation status determined in the pleural effusion fluid and the gefitinib response in the remaining patients and the survival benefit of gefitinib therapy in the patients with *EGFR* mutations are currently being evaluated, and confirmation of the results is expected in the very near future.

In conclusion, our results suggest that the DNA in pleural effusion fluid can be used to detect *EGFR* mutations and that the *EGFR* mutation status determined may be useful as a predictive factor of response to gefitinib.

## Figures and Tables

**Table 1:** Patient characteristics and *EGFR* mutation status

		**EGFR mutation**
	**(n)**	**(n)**
No. of patients	43	11 (25.6%)
*Age (years)*		
Median	63	
Range	39–82	
		
*Gender*		
Male	22	4 (18.2%)
Female	21	7 (33.3%)
		
*Smoking habit*		
Current	9	2 (22.2%)
Former	16	2 (12.5%)
Never	18	7 (38.9%)
		
*Histology*		
Adenocarcinoma	39	11 (28.2%)
Squamous cell carcinoma	1	0 (0%)
Large cell carcinoma	1	0 (0%)
Unclassified	2	0 (0%)
		
*No. of patients treated with gefitinib*	27	8 (29.6%)
PR	7	7 (14.3%)
SD	7	1 (0%)
PD	13	0 (0%)

EGFR=epidermal growth factor receptor; PD=progressive disease; PR=partial response; SD=stable disease.

**Table 2:** Site of mutations in exons 18–21 of *EGFR*

**Nucleotide changes**	**Amino-acid changes**	**No. of patients**
2481_2495del	E746_A750del	6
2482_2496del	E746_A750del	1
2483_2497del	E746_T753del insA	1
2486_2500del	L747_T751del	1
2819T>G	L858R	2

EGFR=epidermal growth factor receptor; del=deletion; ins=insertion.

The numbering of the mutation sites was based on NM_005228.3 (nucleotide) and NP_005219.2 (amino acid).

**Table 3:** Frequency of *EGFR* mutations in DNA from the pleural effusion fluid of NSCLC patients according to (A) gender, (B) histology, (C) smoking habit, and (D) response to gefitinib

**(A) Gender and *EGFR* mutation status**			
	***EGFR* mutation**		
	+	–	
Female	7	14	
Male	4	18	*P*=0.310
			
**(B) Histology and *EGFR* mutation status**			
	***EGFR* mutation**		
	+	−	
Ad	11	28	
Non-Ad	0	4	*P*=0.558
			
**(C) Smoking habit and *EGFR* mutation status**			
	***EGFR* mutation**		
	+	−	
Never	7	11	
Current/former	4	21	*P*=0.156

Ad=adenocarcinoma; EGFR=epidermal growth factor receptor; +=mutation-positive; −=no mutations.

(A)(B)(C); a total of 43 samples were evaluated.

**Table 4:** *EGFR* mutation status in patients who received gefitinib therapy

**Age (years)**	**Gender**	**Smoking**	**Histology**	**EGFR mutation status**	**Response to gefitinib**
62	F	Never	Ad	E747_P753insS	PR
58	F	Never	Ad	E746_A750del	PR
80	F	Never	Ad	E746_A750del	PR
61	M	Never	Ad	E746_A750del	PR
65	M	Former	Ad	E746_A750del	PR
60	M	Current	Ad	E746_A750del	PR
66	F	Never	Ad	E747_T750del	PR
					
76	F	Never	Ad	Wild	SD
57	F	Former	Ad	Wild	SD
40	F	Never	Ad	Wild	SD
72	F	Never	Ad	Wild	SD
58	F	Former	Ad	Wild	SD
66	F	Never	Ad	Wild	SD
65	F	Former	Ad	L858R	SD
					
39	F	Never	Ad	Wild	PD
69	M	Former	Ad	Wild	PD
72	F	Never	Ad	Wild	PD
74	F	Never	Ad	Wild	PD
67	M	Former	Ad	Wild	PD
62	M	Former	SCC	Wild	PD
59	F	Current	Ad	Wild	PD
77	M	Current	Ad	Wild	PD
82	F	Never	Ad	Wild	PD
66	F	Never	Ad	Wild	PD
56	M	Current	Ad	Wild	PD
61	M	Former	Ad	Wild	PD
65	M	Former	Ad	Wild	PD

Ad=adenocarcinoma; EGFR=epidermal growth factor receptor; F=female; M=male; NSCLC=unclassified NSCLC; PD=progressive disease; PR=partial response; SCC=squamous cell carcinoma; SD=stable disease.
